# Gender Differences in Dementia Spousal Caregiving

**DOI:** 10.1155/2012/162960

**Published:** 2012-09-30

**Authors:** Minna Maria Pöysti, Marja-Liisa Laakkonen, Timo Strandberg, Niina Savikko, Reijo Sakari Tilvis, Ulla Eloniemi-Sulkava, Kaisu Hannele Pitkälä

**Affiliations:** ^1^The Social Insurance Institution of Finland and Unit of Primary Health Care, Helsinki University Central Hospital, P.O. Box 20, 00014 University of Helsinki, Helsinki, Finland; ^2^Unit of Primary Health Care, Helsinki University Central Hospital and City of Helsinki Health Center, P.O. Box 20, 00140 University of Helsinki, Helsinki, Finland; ^3^Geriatric Clinic, Department of General Internal Medicine and Geriatric, Helsinki University Central Hospital and Institute of Health Sciences and Geriatrics, University of Oulu, P.O. Box 5000, 90014 Oulu, Finland; ^4^Unit of Primary Health Care, Helsinki University Central Hospital and Department of General Practice, University of Helsinki, P.O. Box 20, 00140 University of Helsinki, Helsinki, Finland; ^5^Geriatric Clinic, Department of General Internal Medicine and Geriatric, Helsinki University Central Hospital, P.O. Box 20, 00140 University of Helsinki, Helsinki, Finland; ^6^Palmenia Centre for Continuing Education, University of Helsinki, P.O. Box 58, 00014 University of Helsinki, Helsinki, Finland

## Abstract

The proportion of male caregivers is rapidly increasing. However, there are few large scale studies exploring gender differences in the burden or coping with caregiving. We investigated this among caregivers of patients with dementia. The study cohort consisted of 335 dyads of wife-husband couples from two studies including dementia patients and their spousal caregivers. Baseline mini-mental state examination (MMSE), clinical dementia rating scale (CDR), neuropsychiatric inventory (NPI), cornell depression scale and charlson comorbidity index (CCI) were used to describe patients with dementia, Zarit burden scale and geriatric depression scale were used to measure experienced burden and depression of caregivers. Mean age of caregivers was 78 years. There were no differences in depression, satisfaction with life, or loneliness according to caregivers' gender. Male caregivers had more comorbidities than females (CCI 1.9 versus 1.1, *P* < 0.001), and the wives of male caregivers had a more severe stage of dementia than husbands of female caregivers (CDR, *P* = 0.048; MMSE14.0 versus 17.7, *P* < 0.001). However, the mean Zarit burden scale was significantly lower among male than female caregivers (31.5 versus 37.5; *P* < 0.001). Lower education of male caregivers tended to be associated with less experienced burden. In conclusion, male caregivers of dementia experienced lower burden than female caregivers despite care recipients' more severe disease.

## 1. Introduction 

Alzheimer's disease and other dementias are the main reasons for need of caregiving at home and for permanent institutional care, and, therefore, they are among the most expensive diseases for our society [1]. Caregivers' high burden has been examined in a number of studies on dementia [2, 3]. Our society is changing—while caregiving has traditionally been considered as women's activity [4], the number of male caregivers is rapidly increasing. In fact, men over 65 years in the UK soon outnumber women as caregivers [4, 5]. Women and men may approach their caregiving differently. Whereas men seem to consider it more as a task, women may take it more comprehensively [4, 6, 7]. Coping strategies such as active problem-solving skills are beneficial in caregiving irrespective of caregiver gender [8].

A review of 93 articles on gender differences in caregiving studies [4] reports that most studies conclude that women experience more distress and use more services than men. However, in respect to worrying and stress, studies have had contradictory findings. Furthermore, several studies have reported no gender differences [4, 9]. Ten studies have focused on male caregivers and characteristics that predict their distress or coping [4, 9]. All these studies have been cross-sectional, with limited number of participants, and eight being qualitative. Only two studies used quantitative analysis of a structured interview data on males [9, 10]. Thus, we lack knowledge on gender differences of continuous caregiving affecting distress and wellbeing. Therefore, we compared the characteristics and burden of male and female spousal caregivers of patients with dementia.

## 2. Materials and Methods

The present study cohort included 335 dyads of wife-husband married couples from two intervention trials (Family Care as Collaboration trial [11] and FINnish Alzheimer EXercise [FINALEX] trial [12]) using the same measures to characterize participants. In the dyads, one of the spouses was suffering from dementia (care recipient) and the other was the caregiver. There were no differencies in the caregivers' burden between the intervention and control groups of the two original trials. In the family care as collaboration trial, 125 dyads were randomized into a multicomponent intervention program (*N* = 63) (including case coordinator, geriatric consultations when needed, support groups for caregivers, and tailored services for two years), or in a control group (*N* = 62). In the FINALEX trial 210 dyads were randomized to a group-based exercise (*N* = 70) or tailored home-based exercise of the demented spouse (*N* = 70), while 70 dyads served as controls. The intervention lasted for one year, with final followup until two years. The baseline data of these trials were used to study gender differences of caregivers and care recipients. All demographic data and medical diagnoses of the dyads were confirmed both from medical records, comorbidity was assessed by the Charlson comorbidity index (CCI), a weighted index taking into account the number and severity of comorbid conditions [13]. At baseline, patients with dementia were assessed with the clinical dementia rating scale (CDR) [14] for functioning and the stage of dementia, and with the mini-mental state examination (MMSE) [15] for cognitive status. The neuropsychiatric inventory (NPI) [16] was used to describe neuropsychiatric symptoms and the Cornell depression scale for mood [17]. Among caregivers, the Zarit burden Scale [18] was used to measure experienced burden (high burden >40 points) and the geriatric depression scale (GDS) [19] to measure depression. Also the one-year follow-up Zarit points among males and females are reported. Caregivers' life satisfaction was inquired by question “Are you satisfied with life?” (yes/no). Experience of loneliness was inquired by question “Do you suffer from loneliness?” (never/sometimes/often or always) and those responding “sometimes” or “often or always” were categorized as suffering from loneliness. All data were collected by experienced study nurses. The Ethics Committee of Internal Medicine of the Hospital District of Helsinki and Uusimaa, Finland approved the protocol.

### 2.1. Statistical Analyses

Males and females were compared with *χ* ^2^-test for categorical variables and Mann-Whitney test for nonnormally distributed continuous variables. Logistic regression analysis was used to determine the independent value of gender on experienced burden in caregiving. Both caregivers' and care recipients' age and CCI, caregivers' education and use of home care services, and care recipients' MMSE points, NPI points and Cornell points were used as covariates in the analyses.

## 3. Results

Altogether 335 husband-wife dyads were examined. At baseline the mean age of both the caregivers and dementia patients was 78 years. The age of male and female caregivers was similar, but the husbands of female caregivers were slightly older that the wives of male caregivers. Only 13.3% of male and 11.6% of female caregivers used home care services (*P* = 0.65). Male caregivers had significantly more comorbidity than female caregivers (CCI 1.9 versus 1.1, *P* < 0.001). However, the female caregivers experienced a significantly higher burden than males according to the Zarit burden scale (37.5 versus 31.5, *P* < 0.001) and females also had higher points in GDS depression scale than males (8.8 versus 7.0, *P* = 0.0025). There were no significant differences between genders in life satisfaction nor in experience of loneliness (Table 1).

The patients with dementia differed in several ways. The female patients suffered from significantly more severe dementia according to CDR and their mean MMSE points were significantly lower than female caregivers' care recipients' points. There were no differences in respect to care recipients' depressive symptoms according to Cornell scale, neuropsychiatric symptoms according to NPI, or comorbidities according to Charlson (Table 1).

In logistic regression analysis adjusted for caregivers' and care recipients' age and CCI, caregivers' education and use of home care services and care recipients' MMSE, NPI, and Cornell scale points, the male gender was protective against high burden (OR 0.33, 95%CI: 0.18 to 0.62; *P* < 0.001). In this analysis, higher NPI points (OR 1.05, 95%CI: 1.03 to 1.08, *P* < 0.001) and higher Cornell points of the care recipient predicted caregivers' high burden (OR 1.07, 95%CI: 1.00 to 1.14, *P* = 0.005). Caregivers' low education tended to be protective against high burden (OR 0.58, 95%CI: 0.31 to 1.08), *P* = 0.08) (Table 2).

The burden of caregiving decreased during the follow-up year in both genders: at 12 months the mean Zarit points were 27.3 (SD 14.4) and 35.9 (SD 15.0) among male and female caregivers, respectively (*P* < 0.001).

## 4. Discussion

Male caregivers having higher level of comorbidities cared for their wives with more severe dementia but did not suffer as much as female caregivers from burden or from depressive symptoms. Even adjusting for various covariates, male gender seemed to be protective when caregiving a person with Alzheimer's disease.

Our study population has been examined very carefully and the diagnosis of dementia was always confirmed by careful diagnostic work-up by geriatricians or neurologists, which is a strength of this study. When compared to other studies, this is by far the largest quantitative study about gender differences in dementia spousal caregiving. Limitation of our study is its cross-sectional nature. Because of the two interventions, where also control group may have some benefit compared to persons not attending any study program, we used only the baseline data in our logistic regression analysis. An intervention effect can be one reason for reduced burden in both genders in one-year follow-up. It can also be assumed that the most severely demented persons with most neuropsychiatric symptoms were dropped-out from our intervention studies because of deaths or hospitalizations during 12 months.

Our quantitative findings are in line with findings of previous, mostly qualitative studies [4]. There are some contradictory findings [9, 20], but most studies report lower level of stress among male caregivers [4, 10, 21]. An unexpected result of lower education associating with lower burden among male caregivers [10] was given tentative support in this larger quantitative study.

Why do males experience lower burden? Some prior studies have suggested that males do not take caregiver's role similarly to females and their coping strategies are different. Studies concerning caregivers' help-seeking behaviour and gender differencies suggest that male caregivers are not as likely as women to be aware of nor to use community services [22]. Males may prefer family independence. In addition, they may perceive that by accepting community services they admit being weak and losing control [23]. In Finland, where most of the women work full-time also outside home, elderly couples often want to handle their own daily life and protect their adult children as far as possible. Quite rare use of home care services is in line with our previous results of 1214 caregivers from year 2005 [24]. 

A large-scale study of 170 patient-spouse dyads concluded that male caregivers' sense of coherence was higher than female caregivers' [25]. Sense of coherence indicates a person's ability to cope with different life situations. The most recent study investigating coping strategies of nine husband caregivers of their cognitively impaired wives [7] identified six strategies these husbands dealt with caregiving: exerting force, focusing on tasks, blocking emotions, minimizing disruption, distracting attention, and self-medicating. Once having a problem-solving method for coping, Kramer [10] also found that less education, satisfaction with one's social participation, and better health were linked to less burden. Why could lower education be associated with lower burden in caregiving? Lower education might be associated with satisfaction with narrower and more simple daily life without any specific events or new experiences in contrary to academic multitasking lifestyle. If you are content with simple life, you do not need to give up with your previous daily habits, which inevitably happens in caregiving a person with dementia. 

Women usually take more responsibility of the well-being of the family from childbearing to care older family members. Considering the usually high emotional responsibility by female nature, it is quite understandable that they have more emotional stress when facing the situation. Cultural aspects also have a great impact on caregiving, possibly women are expected to be more altruistic and more suitable for caregiving than men [2].

Male sex seems to be protective for a family when a caregiver is needed. The coping methods among males, which may lead to this result, are worth of focusing when planning support and services for these families. Coping strategies have been examined in only few studies. They suggest that caregivers reporting high levels of distress appeared more likely to use an emotion-focused coping strategy, for example, wishful thinking, denial, suppressive feelings, self-blaming and avoidance. Caregivers with low levels of distress, in contrast, used problem-solving strategies such as acceptance and instrumental coping [10, 21]. Given the idea that these differences in coping methods are associated with gender differences in dementia caregiving, we could add this aspect also to continuous support program of these families.

## 5. Conclusions

Males experience significantly less burden while taking care of their wives with dementia, irrespective of the more severe stage of cognitive decline of their spouse. These findings should be confirmed and the reasons behind should be explored in further studies.

## Figures and Tables

**Table 1:** Characteristics of caregivers and care recipients with dementia according to gender.

Characteristic	Male caregivers (*N* = 128)	Female caregivers (*N* = 207)	*P* value^1^
Caregivers			
Mean age, years (SD)	77.0 (6.2)	78.4 (5.6)	0.071
Education <8 years, %	33.6	22.7	0.029
Charlson comorbidity index, mean (SD)	1.9 (1.9)	1.1 (1.4)	<0.001
Zarit burden scale, mean (SD)	31.5 (14.9)	37.5 (14.6)	<0.001
GDS, mean (SD)	7.0 (5.2)	8.8 (5.7)	0.0025
Satisfied with life, %	83.1	78.6	0.33
Suffers from loneliness, %	76.8	66.7	0.051

	Female care recipients (*N* = 128)	Male care recipients (*N* = 207)	

Care recipients			
Mean age, years (SD)	76.9 (6.2)	78.4 (5.6)	0.041
MMSE, mean (SD)	14.0 (7.1)	17.7 (6.2)	<0.001
CDR score			
0.5	4.7	2.9	
1	20.3	30.4	0.048
2	50.8	52.5	
3	24.2	14.5	
Charlson comorbidity index, mean (SD)	1.9 (1.6)	2.3 (1.9)	0.17
Cornell, mean (SD)	5.0 (4.3)	6.0 (5.1)	0.099
NPI, mean (SD)	21.9 (13.8)	22.4 (14.7)	0.97
Home care services	13.3	11.6	0.65

Abbreviations: GDS: geriatric depression scale; MMSE: mini-mental state examination; CDR: clinical dementia rating scale; NPI: neuropsychiatric inventory; SD: standard deviation.

^1^Males and females were compared with *χ* ^2^-test for categorical variables and Mann-Whitney test for nonnormally distributed continuous variables.

**Table 2:** Logistic regression analysis exploring independent determinants associated with high burden (Zarit >40 points) in dementia spousal caregiving.

Covariates	OR (Odds ratio)	95% Confidence intervals	*P* value
Caregiver			
Age	1.14	0.73–1.78	0.57
Male gender	0.33	0.18–0.62	<0.001
Education < 8 years	0.58	0.31–1.08	0.09
Cornell depression scale	1.07	1.00–1.14	0.03
Using home care services	1.98	0.93–4.22	0.08
Charlson comorbidity index	1.14	0.96–1.36	0.12
Care recipient			
Age	0.85	0.54–1.32	0.46
MMSE	0.96	0.92–1.00	0.07
NPI	1.05	1.03–1.08	<0.001
Charlson comorbidity index	1.02	0.87–1.19	0.84
Cornell	1.07	1.00–1.14	0.03

Abbreviations: MMSE: mini-mental state examination; NPI: neuropsychiatric inventory.
